# Distribution of bat species in Western Asia: Occurrence records from the Western Asia Bat Research Network (WAB-Net) project

**DOI:** 10.3897/BDJ.12.e132199

**Published:** 2024-08-21

**Authors:** Kendra Phelps, Zahran Al Abdulasalam, Nisreen Al-Hmoud, Shahzad Ali, Mumen Alrwashdeh, Attaullah, Rasit Bilgin, Astghik Ghazaryan, Luke Hamel, Nijat Hasanov, Ioseb Natradze, George Papov, Ketevan Sidamonidze, Andrew Spalton, Lela Urushadze, Kevin J Olival

**Affiliations:** 1 EcoHealth Alliance, New York City, United States of America EcoHealth Alliance New York City United States of America; 2 Environment Authority, Muscat, Oman Environment Authority Muscat Oman; 3 Bio-Safety and Bio-Security Centre, Royal Scientific Society, Amman, Jordan Bio-Safety and Bio-Security Centre, Royal Scientific Society Amman Jordan; 4 Princess Sumaya University for Technology, Amman, Jordan Princess Sumaya University for Technology Amman Jordan; 5 Department of Wildlife & Ecology, University of Veterinary and Animal Sciences, Lahore, Pakistan Department of Wildlife & Ecology, University of Veterinary and Animal Sciences Lahore Pakistan; 6 Institute of Environmental Sciences, Bogazici University, Istanbul, Turkiye Institute of Environmental Sciences, Bogazici University Istanbul Turkiye; 7 Department of Zoology, Yerevan State University, Yerevan, Armenia Department of Zoology, Yerevan State University Yerevan Armenia; 8 Institute of Zoology, Ministry of Science and Education, Baku, Azerbaijan Institute of Zoology, Ministry of Science and Education Baku Azerbaijan; 9 Institute of Zoology, Ilia State University, Tbilisi, Georgia Institute of Zoology, Ilia State University Tbilisi Georgia; 10 R. Lugar Center, National Center for Disease Control and Public Health, Tbilisi, Georgia R. Lugar Center, National Center for Disease Control and Public Health Tbilisi Georgia; 11 Wildlife consultant, Muscat, Oman Wildlife consultant Muscat Oman

**Keywords:** Chiroptera, Middle East, West Asia, biodiversity, mammal

## Abstract

**Background:**

Western Asia represents a mixing pot of diverse bat species with distributions spanning across other geographic regions. Yet, relative to other regions, there is a significant gap in coordinated bat research in Western Asia, thereby resulting in a relatively limited number of curated occurrence records.

**New information:**

The Western Asia Bat Research Network (WAB-Net) project was created to strengthen research capacity and knowledge of the diversity and distribution of bat species in a little-studied region, as well as to collect data to characterise the diversity and prevalence of coronaviruses in diverse bat species. Over a four-year period (2018–2022), we documented 4,278 individual records for 41 bat species using a cross-sectional survey approach at 50 sites in seven Western Asian countries, specifically Armenia, Azerbaijan, Georgia, Jordan, Oman, Pakistan and Turkiye. At each site, we captured, on average, 90 individual bats (range: 9-131) over multiple consecutive nights and used standardised methods to collect demographic and morphological data from captured individuals. We additionally completed a systematic evaluation of environmental characterisation and human-bat interactions at all 50 sites. Here, we report individual occurrence records and site conditions from this multi-country, multi-year sampling effort.

## Introduction

Western Asia serves as a convergence point for bat species originating from diverse geographic regions, including North Africa, South Asia and Europe, with the majority of these species distributed in more than one region. However, research on bat species in Western Asia is limited in comparison to other geographic regions (Phelps et al. 2019) and primarily concentrated on documenting bat diversity and distribution in a single country (Shehab et al. 2007, Benda et al. 2011, Benda and Uhrin 2019, Abdulrahman et al. 2021) or across neighbouring countries, such as the Arabian Peninsula (Harrison and Bates 1991).

Systematic bat survey data are invaluable to support both conservation and zoonotic disease research efforts (Phelps et al. 2019). In Western Asia, much of the bat locality data are based on historical surveys or museum collections, which were often collected opportunistically and within a more limited geographic scope. The collection of occurrence data from ongoing field surveys using standardised methods is critical to providing a more comprehensive picture of bat species distribution, abundance and diversity. This is especially true today given active and growing threats to bats in most parts of the world (Frick et al. 2020), including factors like climate change that are leading to range shifts and contractions for many species (Festa et al. 2022). At the same time, bats are important reservoir hosts for zoonotic diseases, including several emerging human and livestock viruses. Field-collected occurrence data can improve our understanding of bat-microbial (e.g. viruses, bacteria, protozoa) dynamics and provide information for surveillance efforts and disease risk mitigation (Olival et al. 2017, Sánchez et al. 2022).

## General description

### Purpose

The Western Asia Bat Research Network (WAB-Net) project was created to strengthen research capacity and knowledge of the diversity and distribution of bat species in a little-studied region, as well as to collect data to characterise bat-associated coronaviruses (Phelps et al. 2019). More details on the WAB-Net project are available at www.wabnet.org.

### Additional information

Over a four-year period (2018-2022), we conducted standardised, cross-sectional sampling of bat populations at 50 sites in seven Western Asian countries, specifically Armenia, Azerbaijan, Georgia, Jordan, Oman, Pakistan and Turkiye.

## Sampling methods

### Sampling description

Bats were captured primarily using harp traps and mist nets set in flyways or at the entrance to caves or anthropogenic structures in which bats were roosting. On rare occasions, bats were extracted from crevices with hand-held hoop nets. Trapping began approximately 30 minutes prior to sunset, with trapping duration dependent on capture rate (i.e. to prevent capture of more individuals than can be safely processed in a single night), but on average, nightly trapping duration was 4.8 hours. Captured bats were placed individually in a porous cloth bag and hung in a quiet, dry location away from predators. Standard morphological measurements (i.e. forearm length (mm) and body mass (g)) and demographic information (i.e. sex, age class and reproductive status) of each captured bat were recorded. Expert opinion was relied upon for species identification in the field and, when needed, additional morphological measurements, such as ear length (mm), tail length (mm), hind foot length (mm) and head and body length (mm) along with acoustic recordings, were used to aid in identification. Species identification was later confirmed for a subset of individuals of each species per site from each country via barcoding the cytochrome b gene using previously published methods (Townsen et al. 2008). Bats were immediately released at the original site of capture.

Field team members adhered to biosafety protocols established by the PREDICT Consortium (PREDICT Consortium 2020), including the use of personal protective equipment (i.e. field-dedicated clothing and close-toed shoes that could be disinfected, an N95 respirator mask, double-layer nitrile gloves and eye protection) during the capture, handling and sampling of bats. All methods were approved by the Tufts University Institutional Animal Care and Use Committee (protocols #G2018-01 and #G2020-127) and the USAMRDC Animal Care and Use Review Office (protocol #CT-2017-12).

## Geographic coverage

### Description

**Figure 1.**
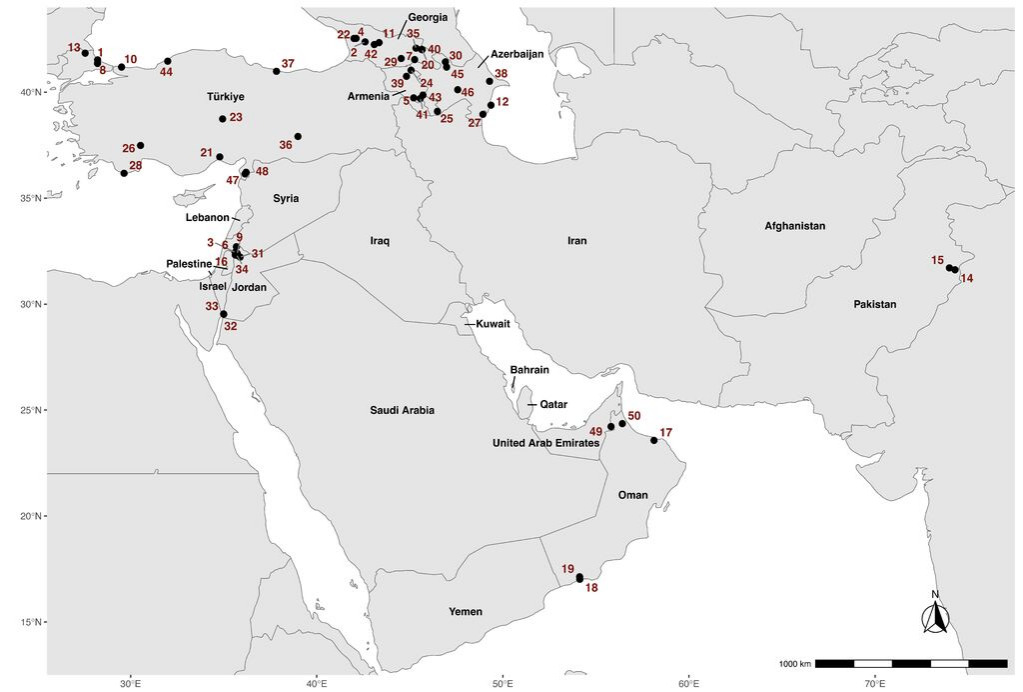
Bats were sampled at 50 sites in seven countries across Western Asia, including Armenia (n = 6 sites), Azerbaijan (n = 6), Georgia (n = 10), Jordan (n = 8), Oman (n = 5), Pakistan (n = 2) and Turkiye (n = 13).

We captured bats at 50 sites in seven Western Asian countries, specifically Armenia, Azerbaijan, Georgia, Jordan, Oman, Pakistan and Turkiye (Fig. 1). The map was created in R version 4.3.2 (R Core Team 2023) using the following packages: "ggrepel" (Slowikowski 2024), "ggspatial" (Dunnington 2023), "rnaturalearth" (Massicotte and South 2023) and "tidyverse" (Wickham et al. 2019).

Locality information and sampling dates for each site are presented in Table 1, with more detailed information about site conditions presented in Suppl. material 3.

## Taxonomic coverage

### Description

We captured 4,278 individual bats of 41 species belonging to nine families, which represents 43.2% of the 95 bat species distributed across the seven focal countries and 35.6% of the 115 bat species documented in Western Asia (Upham et al. 2024). See Suppl. material 1 for detailed information about individual demographic and morphological traits and Suppl. material 2 for final species determination, based on multiple lines of evidence, including DNA barcode results with associated NCBI GenBank accession numbers. It is important to emphasise that, while the cytochrome b gene is extensively used to confirm species identifications, it captures only the maternal elements of the genome (Ballard and Whitlock 2003). Therefore, conclusions drawn solely from short fragments of the mitochondrial DNA may not be reliable enough to correctly differentiate closely related species (Ruedi et al. 2023). Such is the case for two sibling bat species, *Myotisblythii* and *M.myotis* (Furman et al. 2012), which are thought to undergo cryptic hybridisation in geographic areas of sympatry in Western Asia (i.e. Turkiye) (Berthier et al. 2006). Therefore, in addition to DNA barcoding results, which were not informative in some instances, data on morphology, geographic distribution, and/or acoustic recordings was also used to confirm species identifications.

Below, scientific names followed by "*" or "**" indicate a conservation status of "Near Threatened" or "Vulnerable", respectively, as designated by the International Union for Conservation of Nature (IUCN) Red List of Threatened Species (IUCN 2024).

### Taxa included

**Table:** 

Rank	Scientific Name	Common Name
order	Chiroptera	Bats
family	Emballonuridae	Sheath-tailed Bats
species	*Taphozousnudiventris*	Naked-rumped Tomb Bat
species	*Taphozousperforatus*	Egyptian Tomb Bat
family	Hipposideridae	Old World leaf-nosed Bats
species	*Aselliaarabica*	Arabian Trident Leaf-nosed Bat
species	*Aselliatridens*	Geoffroy's Trident Leaf-nosed Bat
family	Miniopteridae	Bent-winged Bats
species	Miniopteruspallidus*	Pallid Long-fingered Bat
species	Miniopterusschreibersii**	Schreibers' Long-fingered Bat
family	Nycteridae	Slit-faced Bats
species	*Nycteristhebaica*	Egyptian Slit-faced Bat
family	Pteropodidae	Old World Fruit Bats
species	*Rousettusaegyptiacus*	Egyptian Rousette
species	Rousettusleschenaultii*	Leschenault's Rousette
family	Rhinolophidae	Horseshoe Bats
species	*Rhinolophusblasii*	Blasius' Horseshoe Bat
species	*Rhinolophusclivosus*	Geoffroy's Horseshoe Bat
species	Rhinolophuseuryale*	Mediterranean Horseshoe Bat
species	*Rhinolophusferrumequinum*	Greater Horseshoe Bat
species	*Rhinolophushipposideros*	Lesser Horseshoe Bat
species	*Rhinolophuslepidus*	Blyth's Horseshoe Bat
species	Rhinolophusmehelyi**	Mehely's Horseshoe Bat
family	Rhinonycteridae	Trident Bats
species	*Triaenopspersicus*	Persian Trident Bat
family	Rhinopomatidae	Mouse-tailed Bats
species	*Rhinopomacystops*	Arabian Mouse-tailed Bat
species	*Rhinopomamicrophyllum*	Greater Mouse-tailed Bat
species	*Rhinopomamuscatellum*	Muscat Mouse-tailed Bat
family	Vespertilionidae	Vesper Bats
species	*Barbastellacaspica*	Caspian Barbastelle
species	Cnephaeus (Eptesicus) serotinus	Eurasian Serotine
species	*Hypsugosavii*	Savi's Pipistrelle
species	*Myotisalcathoe*	Alcathoe Whiskered Myotis
species	*Myotisblythii*	Lesser Myotis
species	Myotiscapaccinii**	Long-fingered Myotis
species	*Myotisdaubentonii*	Daubenton's Myotis
species	*Myotisdavidii*	David's Myotis
species	*Myotisemarginatus*	Geoffroy's Myotis
species	*Myotismyotis*	Greater Myotis
species	*Myotisnattereri*	Natterer's Myotis
species	*Myotistschuliensis*	Tschuli Myotis
species	*Pipistrellusjavanicus*	Javan Pipistrelle
species	*Pipistrelluskuhlii*	Kuhl's Pipistrelle
species	*Pipistrellusnathusii*	Nathusius's Pipistrelle
species	*Pipistrelluspipistrellus*	Common Pipistrelle
species	*Pipistrelluspygmaeus*	Soprano Pipistrelle
species	*Plecotusauritus*	Brown Long-eared Bat
species	*Plecotusmacrobullaris*	Alpine Long-eared Bat
species	*Scotophilusheathii*	Greater Asian Yellow Bat
species	*Scotophiluskuhlii*	Lesser Asian Yellow Bat

## Temporal coverage

### Notes

August 2018 - January 2022

## Usage licence

### Usage licence

Other

### IP rights notes

Usage of reported data is licensed under a Creative Commons Attribution-Non-Commercial 4.0 License (CC BY-NC 4.0).

## Data resources

### Data package title

The Western Asia Bat Research Network (WAB-Net) project

### Resource link

https://www.gbif.org/dataset/7c56c0cb-66e3-4e8f-acb9-db6370c87451

### Number of data sets

1

### Data set 1.

#### Data set name

The Western Asia Bat Research Network (WAB-Net) project

#### Data format

Darwin Core Format (https://dwc.tdwg.org/terms/)

#### Description

Occurrence records for 4,278 individual bats from 41 species captured across 50 sites in seven Western Asian countries (Armenia, Azerbaijan, Georgia, Jordan, Oman, Pakistan and Turkiye) between 2018-2022.

**Data set 1.** 

Column label	Column description
occurrenceID	An identifier for the dwc:Occurrence (as opposed to a particular digital record of the dwc:Occurrence).
basisOfRecord	The specific nature of the data record.
eventDate	The date-time or interval during which a dwc:Event occurred. For occurrences, this is the date-time when the dwc:Event was recorded.
scientificName	The full scientific name, with authorship and date information if known. When forming part of a dwc:Identification, this should be the name in lowest level taxonomic rank that can be determined. This term should not contain identification qualifications, which should instead be supplied in the dwc:identificationQualifier term.
higherClassification	A list (concatenated and separated) of taxon names terminating at the rank immediately superior to the referenced dwc:Taxon.
kingdom	The full scientific name of the kingdom in which the dwc:Taxon is classified.
phylum	The full scientific name of the phylum or division in which the dwc:Taxon is classified.
class	The full scientific name of the class in which the dwc:Taxon is classified.
order	The full scientific name of the order in which the dwc:Taxon is classified.
family	The full scientific name of the family in which the dwc:Taxon is classified.
genus	The full scientific name of the genus in which the dwc:Taxon is classified.
specificEpithet	The name of the first or species epithet of the dwc:scientificName.
taxonRank	The taxonomic rank of the most specific name in the dwc:scientificName.
lifeStage	The age class or life stage of the dwc:Organism(s) at the time the dwc:Occurrence was recorded.
sex	The sex of the biological individual(s) represented in the dwc:Occurrence.
reproductiveCondition	Categorisation of individuals for eusocial species (including some mammals and arthropods).
identifiedBy	A list (concatenated and separated) of names of people, groups or organisations who assigned the dwc:Taxon to the subject.
dateIdentified	The date on which the subject was determined as representing the dwc:Taxon.
decimalLatitude	The geographic latitude (in decimal degrees, using the spatial reference system given in dwc:geodeticDatum) of the geographic centre of a dcterms:Location. Positive values are north of the Equator, negative values are south of it. Legal values lie between -90 and 90, inclusive.
decimalLongitude	The geographic longitude (in decimal degrees, using the spatial reference system given in dwc:geodeticDatum) of the geographic centre of a dcterms:Location. Positive values are east of the Greenwich Meridian, negative values are west of it. Legal values lie between -180 and 180, inclusive.
geodeticDatum	The ellipsoid, geodetic datum or spatial reference system (SRS), upon which the geographic coordinates given in dwc:decimalLatitude and dwc:decimalLongitude are based.
coordinateUncertaintyInMetres	The horizontal distance (in metres) from the given dwc:decimalLatitude and dwc:decimalLongitude describing the smallest circle containing the whole of the dcterms:Location. Leave the value empty if the uncertainty is unknown, cannot be estimated or is not applicable (because there are no coordinates). Zero is not a valid value for this term.
dataGeneralisations	Actions taken to make the shared data less specific or complete than in its original form. Suggests that alternative data of higher quality may be available on request.
georeferencedDate	The date on which the dcterms:Location was georeferenced.
georeferenceSources	A list (concatenated and separated) of maps, gazetteers or other resources used to georeference the dcterms:Location, described specifically enough to allow anyone in the future to use the same resources.
georeferenceVerificationStatus	A categorical description of the extent to which the georeference has been verified to represent the best possible spatial description for the dcterms:Location of the dwc:Occurrence.
higherGeography	A list (concatenated and separated) of geographic names less specific than the information captured in the dwc:locality term.
continent	The name of the continent in which the dcterms:Location occurs.
country	The name of the country or major administrative unit in which the dcterms:Location occurs.
countryCode	The standard code for the country in which the dcterms:Location occurs.
stateProvince	The name of the next smaller administrative region than country (state, province, canton, department, region etc.) in which the dcterms:Location occurs.
county	The full, unabbreviated name of the next smaller administrative region than stateProvince (county, shire, department etc.) in which the dcterms:Location occurs.
municipality	The full, unabbreviated name of the next smaller administrative region than county (city, municipality etc.) in which the dcterms:Location occurs. Do not use this term for a nearby named place that does not contain the actual dcterms:Location.
locality	The specific description of the place.
language	A language of the resource.
licence	A legal document giving official permission to do something with the resource.
institutionID	An identifier for the institution having custody of the object(s) or information referred to in the record.
institutionCode	The name (or acronym) in use by the institution having custody of the object(s) or information referred to in the record.
collectionCode	The name, acronym, coden or initialism identifying the collection or dataset from which the record was derived.
catalogNumber	An identifier (preferably unique) for the record within the dataset or collection.
recordedBy	A list (concatenated and separated) of names of people, groups or organiations responsible for recording the original dwc:Occurrence. The primary collector or observer, especially one who applies a personal identifier (dwc:recordNumber), should be listed first.
preparations	A list (concatenated and separated) of preparations and preservation methods for a dwc:MaterialEntity.
otherCatalogNumbers	A list (concatenated and separated) of previous or alternative fully qualified catalogue numbers or other human-used identifiers for the same dwc:Occurrence, whether in the current or any other dataset or collection.
previousIdentifications	A list (concatenated and separated) of previous assignments of names to the dwc:Organism.
associatedSequences	A list (concatenated and separated) of identifiers (publication, global unique identifier, URI) of genetic sequence information associated with the dwc:MaterialEntity.

## Supplementary Material

E2C00763-AB8E-58D2-AD32-D9B6A0166782 10.3897/BDJ.12.e132199.suppl1 Supplementary material 1 Species occurrence, including demographic and morphological data for each captured individual bat Data type occurrences, morphology, demography Brief description Detailed information about each of the 4,278 individual bats captured during the WAB-Net project, including taxonomic, demographic and morphologic information. File: oo_1105880.csv https://binary.pensoft.net/file/1105880 Kendra Phelps, Zahran Al Abdulasalam, Nisreen Al-Hmoud, Shahzad Ali, Mumen Alrwashdeh, Attaullah, Rasit Bilgin, Astghik Ghazaryan, Luke Hamel, Nijat Hasanov, Ioseb Natradze, George Papov, Ketevan Sidamonidze, Andrew Spalton, Lela Urushadze, Kevin J. Olival

922A07A2-2B07-5A3A-8382-180F187EAC88 10.3897/BDJ.12.e132199.suppl2 Supplementary material 2 Updated species identifications based on multiple lines of evidence Data type cytB barcoding, species identifications Brief description Update of field species identification based on CytB barcoding results, including associated NCBI GenBank accession numbers, geographic distribution, morphology and/or acoustic signatures. File: oo_1112428.csv https://binary.pensoft.net/file/1112428 Kendra Phelps, Zahran Al Abdulasalam, Nisreen Al-Hmoud, Shahzad Ali, Mumen Alrwashdeh, Attaullah, Rasit Bilgin, Astghik Ghazaryan, Luke Hamel, Nijat Hasanov, Ioseb Natradze, George Papov, Ketevan Sidamonidze, Andrew Spalton, Lela Urushadze, Kevin J. Olival

FFD9B05F-EF69-5517-AD73-B29BB189E93E 10.3897/BDJ.12.e132199.suppl3 Supplementary material 3 Environmental conditions and potential bat-human contact interfaces Data type site characteristics Brief description We assessed site-level factors within a 1 km^2^ radius at each of the 50 sites in which bats were sampled, ranging from site type (e.g. forested habitat, wetland, caves and/or rock crevices), potential bat-human interfaces (e.g. tourism, wildlife market) and evidence of human disturbance (e.g. guano harvesting, bat hunting, fire remnants). File: oo_1093057.csv https://binary.pensoft.net/file/1093057 Kendra Phelps, Zahran Al Abdulasalam, Nisreen Al-Hmoud, Shahzad Ali, Mumen Alrwashdeh, Attaullah, Rasit Bilgin, Astghik Ghazaryan, Luke Hamel, Nijat Hasanov, Ioseb Natradze, George Papov, Ketevan Sidamonidze, Andrew Spalton, Lela Urushadze, Kevin J. Olival

## Figures and Tables

**Table 1.** Locality information and sampling dates for sites included in this study. The map code corresponds to sites in Fig. 1. See Suppl. material 3 for more detailed information about each site.

**Map code**	**Country**	**Site**	**Latitude** (North)	**Longitude** (East)	**Sampling start date** (dd/mm/yyyy)	**Sampling end date** (dd/mm/yyyy)
1	Turkiye	Cilingoz Cave	41.52	28.22	21/08/2018	24/08/2018
2	Georgia	Ghliana Cave	42.37	42.60	10/09/2018	12/09/2018
3	Jordan	Pella Cave	32.44	35.62	04/10/2018	06/10/2018
4	Georgia	Letsurtsume Cave	42.54	42.11	07/06/2019	10/06/2019
5	Armenia	Areni 1 Cave	39.73	45.20	12/06/2019	16/06/2019
6	Jordan	Al-Himmah Cave	32.71	35.68	07/07/2019	09/07/2019
7	Georgia	Tetri Senakebi Cave	41.54	45.26	06/08/2019	09/08/2019
8	Turkiye	Yaylacik Cave	41.36	28.22	16/08/2019	18/08/2019
9	Jordan	Baoun Cave	32.39	35.73	22/08/2019	24/08/2019
10	Turkiye	Sofular Cave	41.18	29.51	23/08/2019	27/08/2019
11	Georgia	Samertskhle Klde	42.34	43.35	03/09/2019	05/09/2019
12	Azerbaijan	Fish Reproduction Plant	39.39	49.36	07/09/2019	09/09/2019
13	Turkiye	Dupnisa Cave	41.84	27.56	08/09/2019	11/09/2019
14	Pakistan	Noorjahan & Jahangir Tombs	31.62	74.29	15/10/2019	16/10/2019
15	Pakistan	Sheikhpura Fort	31.71	73.99	18/10/2019	19/10/2019
16	Jordan	Khirbat Al Wahadinah	32.32	35.62	26/10/2019	01/11/2019
17	Oman	Muscat Al Khoud Fort	23.57	58.12	03/11/2019	04/11/2019
18	Oman	Alhaseela Farm	17.01	54.13	01/03/2020	02/03/2020
19	Oman	Suhoor Cave	17.13	54.12	03/03/2020	05/03/2020
20	Georgia	Gremi	42.00	45.66	13/07/2020	16/07/2020
21	Turkiye	Saykoy Cave	36.95	34.79	03/08/2020	05/08/2020
22	Georgia	Becho Cave	42.54	42.00	07/08/2020	12/08/2020
23	Turkiye	Sarihidir Tunnel	38.74	34.94	08/08/2020	10/08/2020
24	Armenia	Arakelots Vanq	41.03	45.07	11/08/2020	13/08/2020
25	Armenia	Shikahogh Mine	39.09	46.48	15/08/2020	17/08/2020
26	Turkiye	Sefer Yitigi Cave	37.48	30.53	17/08/2020	20/08/2020
27	Azerbaijan	Baligchilar Village	38.95	48.92	20/08/2020	22/08/2020
28	Turkiye	Hidirellez Cave	36.17	29.64	22/08/2020	25/08/2020
29	Georgia	Algeti Water Reservoir	41.59	44.53	28/08/2020	02/09/2020
30	Azerbaijan	Gakh	41.43	46.91	09/09/2020	11/09/2020
31	Jordan	King Talal Dam	32.22	35.88	01/10/2020	01/10/2020
32	Jordan	Aqaba Castle	29.52	35.00	12/10/2020	12/10/2020
33	Jordan	Saraya-Aqaba	29.54	34.99	13/10/2020	13/10/2020
34	Jordan	Kufranjah Cave	32.30	35.70	21/10/2020	22/10/2020
35	Georgia	Pichkhovani Church	42.08	45.33	21/06/2021	22/06/2021
36	Turkiye	Firat	37.91	38.99	07/07/2021	08/07/2021
37	Turkiye	Kahyaoglu	40.99	37.83	13/07/2021	14/07/2021
38	Azerbaijan	Jangi Gobustan	40.51	49.28	15/07/2021	17/07/2021
39	Armenia	Dilijan	40.75	44.82	29/07/2021	31/07/2021
40	Georgia	Grdzeli Chala	42.02	45.62	31/07/2021	07/08/2021
41	Armenia	Getashen	39.70	45.56	03/08/2021	05/08/2021
42	Georgia	Vardigora	42.25	43.09	03/08/2021	09/08/2021
43	Armenia	Jermuk	39.86	45.70	06/08/2021	09/08/2021
44	Turkiye	Cayirkoy Cave	41.46	31.99	10/08/2021	11/08/2021
45	Azerbaijan	Shaki	41.18	46.98	23/08/2021	25/08/2021
46	Azerbaijan	Aghjabadi	40.12	47.57	10/09/2021	12/09/2021
47	Turkiye	Harbiye Cave	36.14	36.14	22/09/2021	26/09/2021
48	Turkiye	Narlica Cave	36.22	36.20	27/09/2021	28/09/2021
49	Oman	Saarani Falaj	24.22	55.81	16/01/2022	18/01/2021
50	Oman	Baidha Tunnel	24.36	56.42	19/01/2022	20/01/2022
